# Glucocorticoid stimulation induces regionalized gene responses within topologically associating domains

**DOI:** 10.3389/fgene.2023.1237092

**Published:** 2023-07-27

**Authors:** Christophe Tav, Éric Fournier, Michèle Fournier, Fatemeh Khadangi, Audrey Baguette, Maxime C. Côté, Maruhen A. D. Silveira, Félix-Antoine Bérubé-Simard, Guillaume Bourque, Arnaud Droit, Steve Bilodeau

**Affiliations:** ^1^ Centre de Recherche du CHU de Québec—Université Laval, Axe Oncologie, Québec, QC, Canada; ^2^ Centre de Recherche sur le Cancer de l’Université Laval, Québec, QC, Canada; ^3^ Centre de Recherche en Données Massives de l’Université Laval, Québec, QC, Canada; ^4^ Department of Human Genetics, Faculty of Medicine, McGill University, Montréal, QC, Canada; ^5^ Canadian Center for Computational Genomics, McGill University, Montréal, QC, Canada; ^6^ Centre de Recherche du CHU de Québec—Université Laval, Axe Endocrinologie et Néphrologie, Québec, QC, Canada; ^7^ Département de Médecine Moléculaire, Faculté de Médecine, Université Laval, Québec, QC, Canada; ^8^ Département de Biologie Moléculaire, Biochimie Médicale et Pathologie, Faculté de Médecine, Université Laval, Québec, QC, Canada

**Keywords:** transcription, position effects, coregulators, topologically associating domains, glucocorticoid receptor

## Abstract

Transcription-factor binding to cis-regulatory regions regulates the gene expression program of a cell, but occupancy is often a poor predictor of the gene response. Here, we show that glucocorticoid stimulation led to the reorganization of transcriptional coregulators MED1 and BRD4 within topologically associating domains (TADs), resulting in active or repressive gene environments. Indeed, we observed a bias toward the activation or repression of a TAD when their activities were defined by the number of regions gaining and losing MED1 and BRD4 following dexamethasone (Dex) stimulation. Variations in Dex-responsive genes at the RNA levels were consistent with the redistribution of MED1 and BRD4 at the associated cis-regulatory regions. Interestingly, Dex-responsive genes without the differential recruitment of MED1 and BRD4 or binding by the glucocorticoid receptor were found within TADs, which gained or lost MED1 and BRD4, suggesting a role of the surrounding environment in gene regulation. However, the amplitude of the response of Dex-regulated genes was higher when the differential recruitment of the glucocorticoid receptor and transcriptional coregulators was observed, reaffirming the role of transcription factor-driven gene regulation and attributing a lesser role to the TAD environment. These results support a model where a signal-induced transcription factor induces a regionalized effect throughout the TAD, redefining the notion of direct and indirect effects of transcription factors on target genes.

## Introduction

Gene regulation is controlled by the recruitment of transcriptional regulators to cis-regulatory regions and implicates an intricate interplay with the chromatin environment and the chromosome architecture (Lee and Young, 2013; Kim and Shendure, 2019; Schoenfelder and Fraser, 2019). In fact, the environment surrounding a gene is an important determinant of its transcriptional regulation as exemplified by the gene position effect (Elgin and Reuter, 2013). Furthermore, the use of transgenes in animal models highlighted that the integration site is a major determinant of expression levels. Random integration of transgenes in the mouse genome confirmed large differences in transcription levels depending on the integration site (Akhtar et al., 2013). In fact, the transgene typically adopts the chromatin environment of the integration site, which will influence gene regulation (Akhtar et al., 2013; Despang et al., 2019). However, why and how this process takes place remains poorly understood.

In higher eukaryotes, genes are clustered not only in the linear genome but also in the three-dimensional genome (Bonev and Cavalli, 2016; Rowley and Corces, 2018; Schoenfelder and Fraser, 2019; Misteli, 2020). Indeed, physical interactions between genomic regions create a multilevel structure. At high levels, compartments A and B segregate actively transcribed regions from repressed regions (Lieberman-Aiden et al., 2009; Dixon et al., 2012; Wang et al., 2016). At a smaller scale, topologically associating domains (TADs) represent self-interacting regions favoring contacts between cis-regulatory regions and genes (Lieberman-Aiden et al., 2009; Nora et al., 2012; Wang et al., 2016). In addition to physical proximity between genes, there is an expanding body of evidence suggesting that genes are transcriptionally co-regulated in response to external signals (Osborne et al., 2004; Fanucchi et al., 2013). In fact, genes belonging to the same TAD are often co-expressed (Le Dily et al., 2014; Boudaoud et al., 2017; Hsieh et al., 2020; Krietenstein et al., 2020). For instance, activated and repressed genes following steroid stimulation are often segregated into distinct TADs (Le Dily et al., 2014; Vockley et al., 2016; D’Ippolito et al., 2018). In addition to TADs, smaller structures such as sub-TADs (Rao et al., 2014; Rowley et al., 2017; Huang et al., 2019) and co-expression domains (Osborne et al., 2004; Soler-Oliva et al., 2017) have also been supporting the notion of gene co-regulation. Yet, the mechanisms driving gene co-regulation in response to external signals are still unclear.

Transcription factors modify the transcriptional program in response to external signals by converging toward sequence-specific DNA motifs within cis-regulatory regions. Accordingly, they play major roles in normal and disease development by establishing and maintaining cell states (Lee and Young, 2013). Across the genome, transcription factors bind in clusters and synergize to control the transcriptional program (Spitz and Furlong, 2012; Voss and Hager, 2015; Liu and Tjian, 2018). Transcription factors and their coregulators form condensates, creating dynamic environments surrounding active genes (Hnisz et al., 2017; Boija et al., 2018; Cho et al., 2018; Chong et al., 2018; Sabari et al., 2018; Zamudio et al., 2019). Furthermore, it is generally accepted that physical proximity between enhancer and promoter regions is responsible for transmitting the effect of distally bound transcription factors to promoter regions (Deng et al., 2012; 2014; Lee et al., 2015; Chen et al., 2018). However, transcriptional changes are often observed without evidence of direct occupancy by the induced transcription factors or proximity of the cis-regulatory regions with the gene promoter (Alexander et al., 2019; Benabdallah et al., 2019; Schoenfelder and Fraser, 2019). These observations suggest that transcription factors create a regionalized impact on a gene domain through modulation of coregulators and/or the chromosome architecture.

Signal-induced transcription factor stimulations, including steroid nuclear receptors, are associated with a global redistribution of transcriptional coregulators, leading to the activation or repression of specific genes (López-Maury et al., 2008; Voss and Hager, 2015). Among them, the glucocorticoid receptor (GR, NR3C1) leads to the rapid activation and repression of target genes (Reddy et al., 2009). Interestingly, gene activation and repression are not always correlated with GR DNA occupancy (Thormann et al., 2018), suggesting the implication of alternative mechanisms involved in gene regulation. Considering that the GR interacts with a large number of transcriptional coregulators, activation and repression mechanisms were suggested to involve competition mechanisms with other transcription factors (Schmidt et al., 2016). Since, GR-responsive genes are spatially connected through pre-stimulation interactions (D’Ippolito et al., 2018; Portuguez et al., 2022), an interesting possibility is that signal-induced transcription factors, when activating and repressing, modulate the microenvironment within a gene domain leading to co-regulated transcriptional changes.

Here, we show that glucocorticoids, through the GR, induce a reorganization of transcriptional coregulators MED1 and BRD4. These regionalized changes in gene regulation are secluded within TADs, showing gains and losses in MED1 and BRD4. The presence of a gene within a changing TAD is an important determinant of the gene response. Our model proposes that regionalized gene regulation within TADs is a direct consequence of the modulation of the levels of transcriptional coregulators in response to glucocorticoids.

## Materials and methods

### Cell culture

A549 (ATCC, CCL-185) cells were grown in F12K medium (Gibco, 21127022). The culture medium was supplemented with 10% fetal bovine serum (Invitrogen, qualified 12483020), 100 μM MEM non-essential amino acids (Cellgro, 25-0250), 2 mM L-glutamine (Gibco, 25030-081), 100 U/mL penicillin, and 100 μg/mL streptomycin (Gibco, 15170-063). For Dex (Sigma, D1756) stimulations, cells were kept for 3 days in phenol red-free DMEM (Corning, #17-205-CV) supplemented with 5% charcoal/dextran-stripped fetal bovine serum (Fisher, #SH3006803), 100 μM MEM non-essential amino acids, 2 mM L-glutamine, 100 U/mL penicillin, and 100 μg/mL streptomycin. A Dex concentration of 100 nM was used for 60 min.

### ChIP-seq

ChIP-seq experiments were performed as described previously (Bilodeau et al., 2009; Kagey et al., 2010; Fournier et al., 2016; Boudaoud et al., 2017). In brief, 50 million cells were cross-linked for 10 min with 1% formaldehyde and quenched with 125 mM glycine for 5 min. Cells were then washed with PBS, pelleted, flash frozen, and stored at −80°C. Sonicated DNA fragments were immunoprecipitated with antibodies directed against MED1 (Bethyl Laboratories, A300-793A) and BRD4 (Bethyl Laboratories, A301-985A50). Library preparation and high-throughput sequencing were performed at the McGill University and Génome Québec Innovation Centre (MUGQIC), Montréal, Canada. Previously published GR ChIP-seq raw data were retrieved from the ENCODE portal. Analysis of raw sequencing reads was performed using the standard analysis pipelines for quality control, enrichment quantification, and visualization from the Canadian Center for Computational Genomics (Bourgey et al., 2019) for ChIP-seq analysis (version 4.1.2). In brief, reads were trimmed using Trimmomatic (Bolger et al., 2014). High-quality reads were aligned to the human reference genome (hg38) using the BWA (Li and Durbin, 2009) aligner. PCR duplicates were removed using Picard’s MarkDuplicates (http://broadinstitute.github.io/picard/). Narrow peaks were called using MACS2 (Zhang et al., 2008) callpeak with the following options: --nomodel --gsize 2479938032.8 and supplying the sequenced corresponding input DNA as the background control. Peaks overlapping with ENCODE DAC exclusion list regions (Amemiya et al., 2019) (accession number ENCSR636HFF) and belonging to non-standard chromosomes were removed. Analysis of read coverages per bins was performed using multiBamSummary from deepTools 3.5.1 (Ramírez et al., 2016). To identify differentially occupied regions, we used the R package DiffBind (Ross-Innes et al., 2012) [using the DESeq2 method (Love et al., 2014)] with the following parameters: summits = TRUE, minOverlaps = 2, and adjusted *p*-value (false discovery rate) < 5%. The volcano plot was generated using the R package EnhancedVolcano. Heatmaps were generated using computeMatrix and plotHeatmap from deepTools 3.5.1. To generate genomic visualizations, reads from BAM files were normalized using reads per kilobase per million mapped reads (RPKM), extended to 225 bp using the bamCoverage function from deepTools 3.5.1 (Ramírez et al., 2016), and uploaded to the University of California, Santa Cruz (UCSC) Genome Browser (Kent et al., 2002). To identify enriched DNA-binding motifs, the CentriMo tool (version 5.5.3) (Bailey and MacHanick, 2012) from MEME suite was used with --local --score 5.0 --ethresh 10.0 parameters as the input and the JASPAR database for non-redundant transcription factor binding sites in eukaryotes (Castro-Mondragon et al., 2022) as reference.

Genomic region–gene associations were performed using the Genomic Regions Enrichment of Annotations Tool implemented in the R package rGREAT (McLean et al., 2010; Gu and Hübschmann, 2023) using “basal plus extension” as a gene regulatory domain definition and a maximum extension of 10 kb. To assign distal cis-regulatory regions to genes, genomic regions were integrated with chromatin interactions from Hi-C data (described in the following section). The ChromHMM 18-state model dataset from human A549 cells following 1 h Dex stimulation was retrieved from the ENCODE consortium website (accession number ENCSR931PHX). The percentages were computed as the proportion of nucleotides overlapping with each chromatin state. The 18 chromatin states are as follows: active transcription start site (TssA), flanking transcription start site (TssFlnk), upstream flanking transcription start site (TssFlnkU), downstream flanking transcription start site (TssFlnkD), strong transcription (Tx), weak transcription (TxWk), genic enhancer 1 (EnhG1), genic enhancer 2 (EnhG2), active enhancer 1 (EnhA1), active enhancer 2 (EnhA2), weak enhancer (EnhWk), ZNF genes and repeats (ZNF/Rpts), heterochromatin (Het), bivalent/poised TSS (TssBiv), bivalent enhancer (EnhBiv), repressed Polycomb (ReprPC), weak repressed Polycomb (ReprPCWk), and Quiescent/low (Quies).

### Chromosome architecture

A549 Hi-C raw data (D’Ippolito et al., 2018; McDowell et al., 2018) were retrieved from the ENCODE portal (Davis et al., 2018) and processed using the HiC-Pro pipeline version 3.1.0 (Servant et al., 2015). In brief, paired-end reads were aligned to the hg38 reference genome using Bowtie 2 (Langmead and Salzberg, 2012), and default parameters were used to remove duplicate and low-map quality reads and assign reads to MboI restriction fragments. Hi-C interaction matrices were generated at a resolution of 50, 10, and 5 kb. Significant chromatin interactions were identified using FitHic2 version 2.0.8 (Kaul et al., 2020) at 5 kb and 10 kb resolution of the interaction matrix in control and Dex (1 h) conditions. The GenomicInteractions R package (Harmston et al., 2015) was used for manipulating chromatin interaction data. TADs were identified at a resolution of 50 kb using Armatus version 2.3 (Filippova et al., 2014) with a gamma parameter of 0.8. For each TAD, the number of regions gaining and losing MED1 and BRD4 was computed to calculate the TAD score using the following formula: gain/(gain + loss).

### Transcriptomic

Previously published Dex-stimulated A549 RNA-seq raw data were retrieved from the ENCODE portal and processed using the MUGQIC RNA-Seq pipeline version 4.1.2 (Bourgey et al., 2019). In brief, reads were trimmed for adaptor sequences using Trimmomatic (Bolger et al., 2014). High-quality reads were aligned to the human reference genome (hg38) using the STAR aligner (Dobin et al., 2013). PCR duplicates were removed using Picard’s MarkDuplicates (http://broadinstitute.github.io/picard/). Gene counts were determined using featureCounts (version 2.0.1) (Liao et al., 2014) with the genomic annotation Ensembl release 104. Samples considered as outliers were removed after the visual inspection of the PCA plot and assessment of the distance between samples. Differentially expressed genes were identified using DESeq2 (Love et al., 2014) and called significant when the Benjamini–Hochberg-corrected *p*-values were under 0.05. Upregulated genes were selected at a minimum log2 fold change > 0.75 and downregulated genes at a minimum log2 fold change < −0.75. A gene was considered activated or repressed if it was selected at least once between 0 and 6 h. A gene was defined as active if having at least one read in 60% of the samples between 0 and 6 h. Genes defined as inactive were not considered in downstream analysis.

## Results

### Glucocorticoid response implicates redistribution of MED1 and BRD4

**FIGURE 1:**
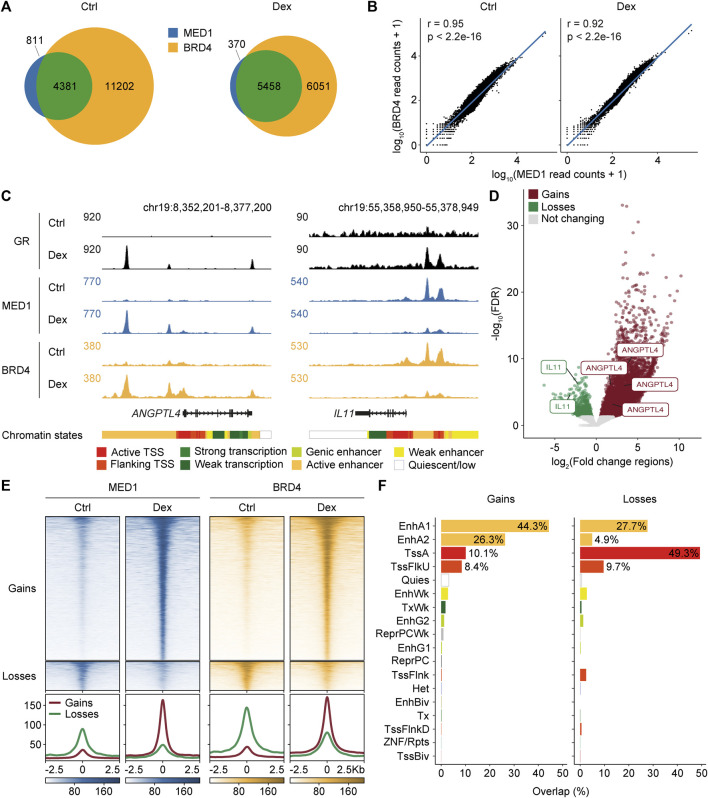
Redistribution of MED1 and BRD4 is correlated across the genome following glucocorticoid stimulation. **(A)** Venn diagram representing MED1 and BRD4 consensus occupied regions in A549 cells in control (Ctrl) and Dex-stimulated conditions. **(B)** Correlations of genome-wide read coverage between MED1 and BRD4 ChIP-seq samples in control and Dex-stimulated conditions. Coverage calculation was performed for consecutive bins of equal size (10 kb). A log10 transformation was applied after adding a pseudocount of 1 to the coregulator read count. Pearson’s correlation was computed using the read count raw values. **(C)** ChIP-seq occupancy profiles of GR, MED1, and BRD4 at *ANGPTL4* and *IL11* genes, which are known to be activated and repressed by glucocorticoids, respectively. ChIP-seq profiles are displayed in RPKM. Gene depictions are presented below the ChIP-seq tracks. The 18-model chromatin states were retrieved from ENCODE and gathered in categories as follows: active TSS (TssA), flanking TSS (TssFlnk, TssFlnkU, and TssFlnkD), strong transcription (Tx), weak transcription (TxWk), genic enhancer (EnhG1 and EnhG2), active enhancer (EnhA1 and EnhA2), weak enhancer (EnhWk), and quiescent/low (Quies). **(D)** Volcano plot of differentially occupied regions combining ChIP-seq replicates for MED1 and BRD4 after a 1-h Dex stimulation. Regions gaining (*n* = 5,442) and losing (*n* = 1,097) MED1 and BRD4 are represented by red and green dots, respectively. Regions associated with *ANGPTL4* and *IL11* are displayed. **(E)** Top—density heatmaps representing MED1 and BRD4 ChIP-seq intensities at regions gaining (*n* = 5,442) and losing (*n* = 1,097) transcriptional coregulators following Dex stimulation in A549 cells. Regions were sorted in descending order based on the mean value per region. Bottom—average ChIP-seq signal for regions gaining and losing MED1 and BRD4. A region of 5 kb centered on the occupied region is displayed. **(F)** Overlaps of regions gaining and losing MED1 and BRD4 with the 18-model chromatin states.

To explore the generation of regional effects on gene regulation, we examined the recruitment of the bromodomain-containing protein 4 (BRD4) and the mediator complex subunit MED1, which are functional coregulators of the GR (Chen and Roeder, 2007; Pradhan et al., 2016) and are found within mobile phase separation droplets (Sabari et al., 2018). Experiments were carried out in A549 cells following a 60-min treatment with Dex. Between 8,189 and 27,310, regions were identified in the different datasets, and the consensus was determined by the overlap between replicates (Supplementary Table S1). As expected, a large proportion of regions occupied by MED1 were shared with BRD4 in control (84.4%) and Dex-stimulated cells (93.7%) (Figure 1A). Accordingly, the read coverage across the genome was highly correlated between the two transcriptional coregulators in control (Pearson’s r = 0.95 and *p* < 2.2e-16) and Dex-stimulated (Pearson’s r = 0.92 and *p* < 2.2e-16) cells (Figure 1B). Concurrent gains and losses in MED1 and BRD4 were frequently observed throughout the genome. For example, regulatory regions of *ANGPTL4* and *IL11* genes, which are positively and negatively Dex-regulated genes, respectively, (S1A-B Fig), showed increased and decreased levels of BRD4 and MED1 at regions bound by the GR (Figure 1C). Overall, concurrent increases and decreases at 5,442 and 1,097 regions, respectively, were identified combining the MED1 and BRD4 replicates (Figure 1D; Supplementary Table S2). These regions will be referred to as regions gaining and losing MED1 and BRD4 thereafter. To confirm that regions integrating replicates for BRD4 and MED1 properly identified differential regions for both coregulators, we generated ChIP-seq density heatmaps (Figure 1E). Increases and decreases in both MED1 and BRD4 were observed in regions defined as gaining and losing transcriptional coregulators. At these regions, the variations in MED1 and BRD4 signals following the Dex stimulation were correlated (for gains, Pearson’s r = 0.94 and *p* < 2.2e-16; for losses, Pearson’s r = 0.91 and *p* < 2.2e-16, Supplementary Figure S1C). Therefore, our results confirm that Dex stimulation reorganizes MED1 and BRD4 genome-wide.

To validate that the GR was directly implicated in the rearrangement of transcriptional coregulators, regions gaining and losing BRD4 and MED1 were overlaid with the GR genome-wide occupancy using the available data (D’Ippolito et al., 2018; McDowell et al., 2018). A large subset of regions gaining (97.8%) and losing (40.7%) MED1 and BRD4 was occupied by the GR (Supplementary Table S2), consistent with a global reorganization of transcriptional coregulators following the direct action of the GR. To functionally assess the genomic regions with the differential recruitment of MED1 and BRD4, we integrated information from the 18-model chromatin states (Ernst and Kellis, 2012) (Figure 1F). Most regions gaining MED1 and BRD4 were associated with enhancers (EnhA1 and EnhA2), while regions losing coregulators were primarily found at promoter (Tss and TssFlnkU) regions. As expected, gains were associated with the GR DNA-binding motif, while losses were enriched for the DNA-binding motif of the AP-1 family of transcription factors (Supplementary Figure S1D). These findings confirm the GR-associated modulation of cis-regulatory regions following Dex stimulation.

### Gains and losses in MED1 and BRD4 correlate with gene level variations

**FIGURE 2:**
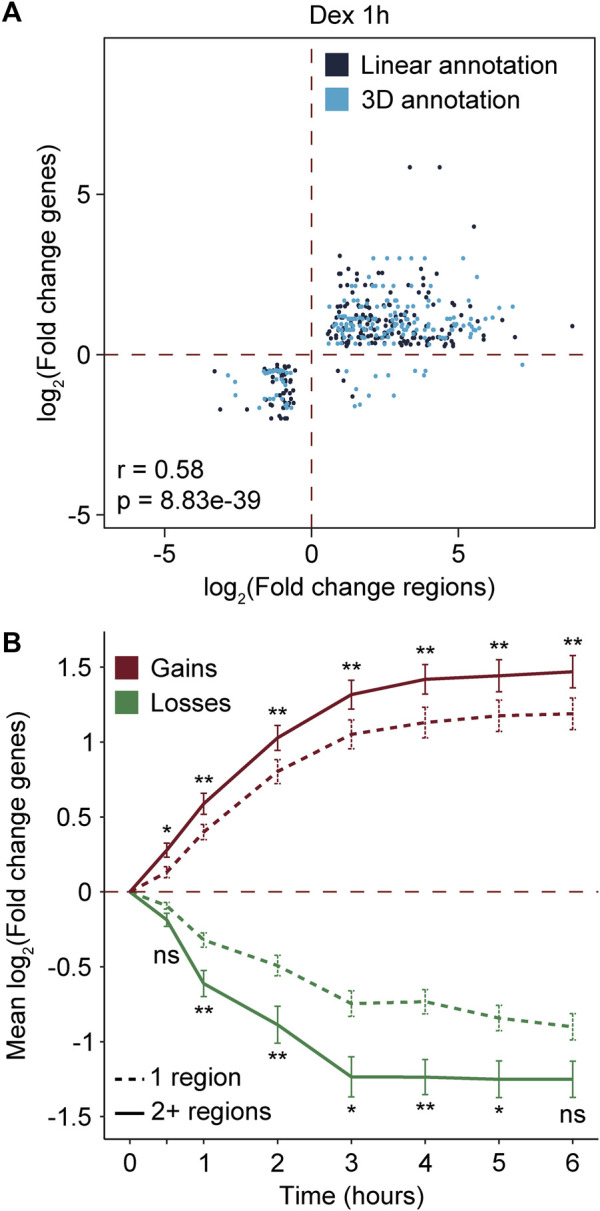
Differential recruitment of MED1 and BRD4 is associated to gene level variations. **(A)** Scatter plot showing the correlation between variations in RNA levels of Dex-regulated genes and the recruitment of MED1 and BRD4 at their cis-regulatory regions after 1 h of Dex stimulation. For RNA levels, the log2 of the fold change is represented. For recruitment of transcriptional coregulators, the log2 of the read density at each differential region is represented. Regions gaining and losing MED1 and BRD4 were associated to genes based on their linear proximity or the presence on 3D chromatin interactions. Correlations were assessed using Pearson’s correlation method. **(B)** Time course analysis of the variations at the RNA level of genes associated with 1 or multiple (2+) regions with the differential recruitment of transcriptional coregulators. For RNA levels, the average of the log2 of the fold change for each gene within the group and the standard error of the mean (error bars) are represented. Mann–Whitney U tests were used to compare means between genes associated with 1 or multiple regions at 0.5, 1, 2, 3, 4, 5, and 6 h. The Benjamini–Hochberg procedure was applied on the empirical *p*-values to correct for multiple testing. ns, not significant; *p < 0.05 and **p < 0.01.

To establish whether the differential recruitment of MED1 and BRD4 was associated with variations at the gene level, we used an available transcriptomic dataset of A549 cells stimulated with Dex (D’Ippolito et al., 2018; McDowell et al., 2018). A total of 1,759 differentially expressed genes (911 activated and 848 repressed) were identified between 0 and 6 h of Dex stimulation (Supplementary Figure S2A; Supplementary Table S3). Regions gaining and losing MED1 and BRD4 following Dex treatment were assigned linearly to genes using rGREAT (McLean et al., 2010; Gu and Hübschmann, 2023) or connected to a gene via chromatin interactions using publicly available Hi-C data (D’Ippolito et al., 2018) (see *Materials and Methods*). As expected, gains in MED1 and BRD4 were enriched in Dex-activated genes (Fisher’s test, OR = 9.3, and *p*-value = 3.58e-164). On the other hand, losses in transcriptional coregulators were enriched in Dex-repressed genes (Fisher’s test, OR = 4.5, and *p*-value = 4.2e-40). To determine if the differential recruitment of MED1 and BRD4 was associated with the amplitude of RNA-level variations, we compared fold changes. Variations in MED1 and BRD4 were correlated with variations at the RNA levels at all Dex stimulation time points (Figure 2A; Supplementary Figure S2B). In addition to differential recruitment, the number of regions gaining and losing MED1 and BRD4 could be a determining factor in the gene output. When genes were associated with two or more regions gaining or losing transcriptional coregulators MED1 and BRD4, the mean change in RNA levels was greater (Figure 2B). The presence of two or more regions with differential recruitment was associated with an increase between 1.23- and 2.12-folds and a decrease between 1.49- and 1.91-folds at the RNA level. Therefore, our results support that the differential recruitment of transcriptional coregulators MED1 and BRD4 at cis-regulatory regions is associated with variations at the gene levels.

### Regional gains and losses in MED1 and BRD4 in response to Dex

**FIGURE 3:**
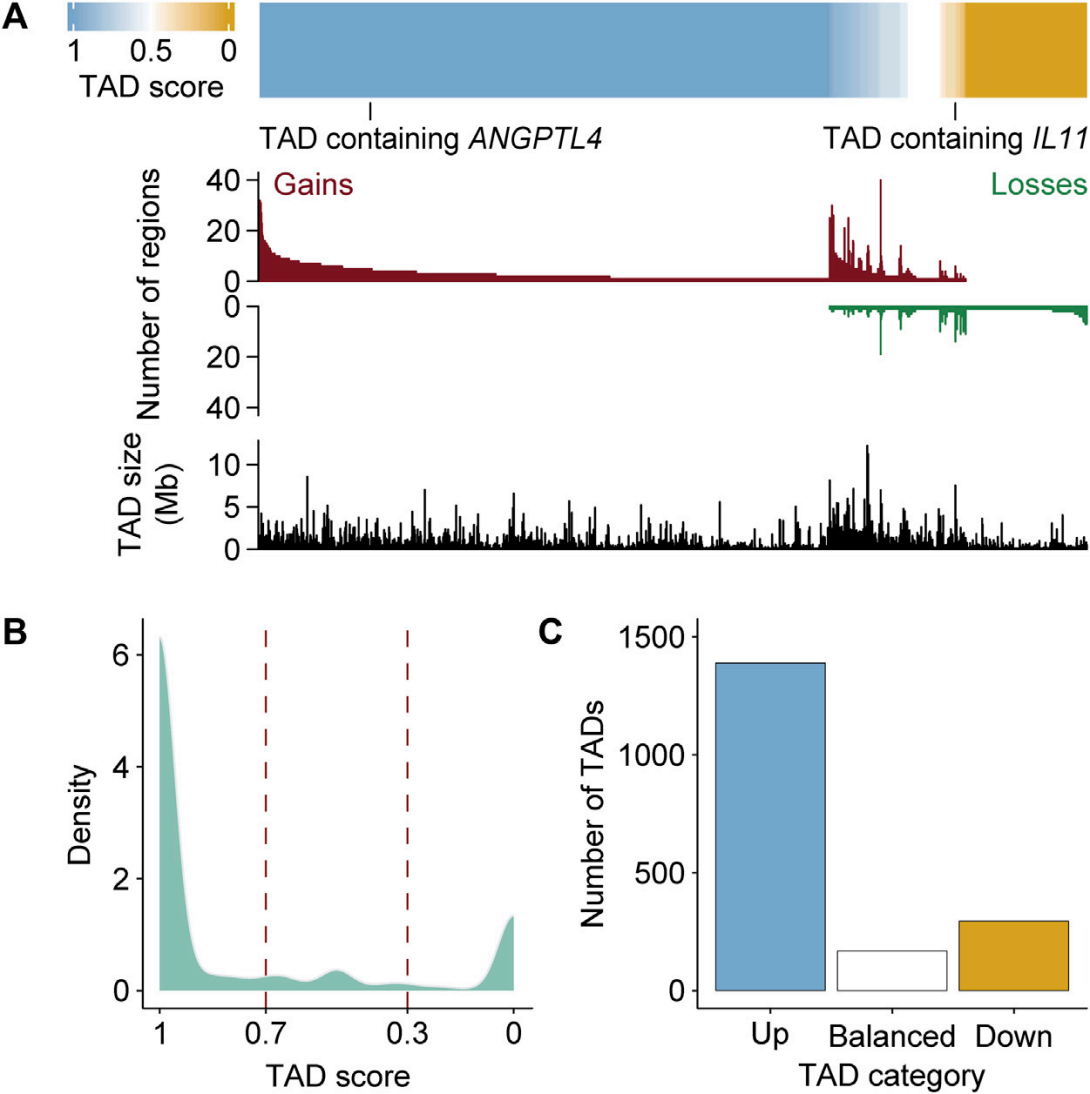
TADs are biased toward gaining or losing MED1 and BRD4 in response to a glucocorticoid stimulation. **(A)** The ratios of regions gaining and losing MED1 and BRD4 within each TAD are biased. The TAD score [= number of gains/(number of gains + losses)] was calculated for each of the 1,851 TADs with at least one region with the differential recruitment of MED1 and BRD4. Top—ranking of TADs based on the score. Values of 0 and 1 are, respectively, equal to a TAD with only regions losing or gaining MED1 and BRD4. Middle—the number of regions gaining and losing MED1 and BRD4 in each TAD is displayed. Bottom—the size of each TAD (Mb) is displayed. **(B)** Density plot representing the bimodal distribution of TAD scores. **(C)** Quantification of the number of TADs defined as up (score ≥ 0.7), balanced (0.3 < score < 0.7), and down (score ≤ 0.3).

The chromosomal architecture is known to delimit different transcriptional activities within the nucleus and influence the range of action of enhancer regions (Dowen et al., 2014; Schoenfelder and Fraser, 2019). Among key factors for chromosome organization, TADs could represent physical frontiers to subdivide gene domains. Furthermore, TADs are maintained following hormonal stimulation and associated with the creation of coordinated regulatory units (Le Dily et al., 2014; D’Ippolito et al., 2018). To directly test the relationship of regions gaining and losing MED1 and BRD4 with TADs, we determined TAD boundaries at 1 h after Dex stimulation in A549 cells by processing available Hi-C data using Armatus (Filippova et al., 2014; D’Ippolito et al., 2018; McDowell et al., 2018) (Supplementary Table S4). Each region differentially occupied by MED1 and BRD4 was assigned to its corresponding TAD, and ratios of gains and losses were calculated [score = (gain)/(gain + loss)] (Figure 3A). Of the 4,957 TADs, 37.3% (1,851 TADs) were associated with at least one region with the differential recruitment of MED1 and BRD4. TAD scores were distributed following a bimodal distribution (Hartigan’s dip test and *p* < 2.2e-16), highlighting a bias toward homogeneity for the differential recruitment of transcriptional coregulators (Figure 3B). Indeed, TADs were biased toward either gaining (75% with a score ≥ 0.7, referred to as up) or losing (16% with a score ≤ 0.3, referred to as down) MED1 and BRD4 (Figure 3C). On average, 3.4 regions with the differential recruitment of MED1 and BRD4 were found among TADs scored as up, down, or balanced. These results support the regionalized recruitment of transcriptional coregulators following stimulation of the GR.

### The TAD environment surrounding a gene influences the response to Dex

**FIGURE 4:**
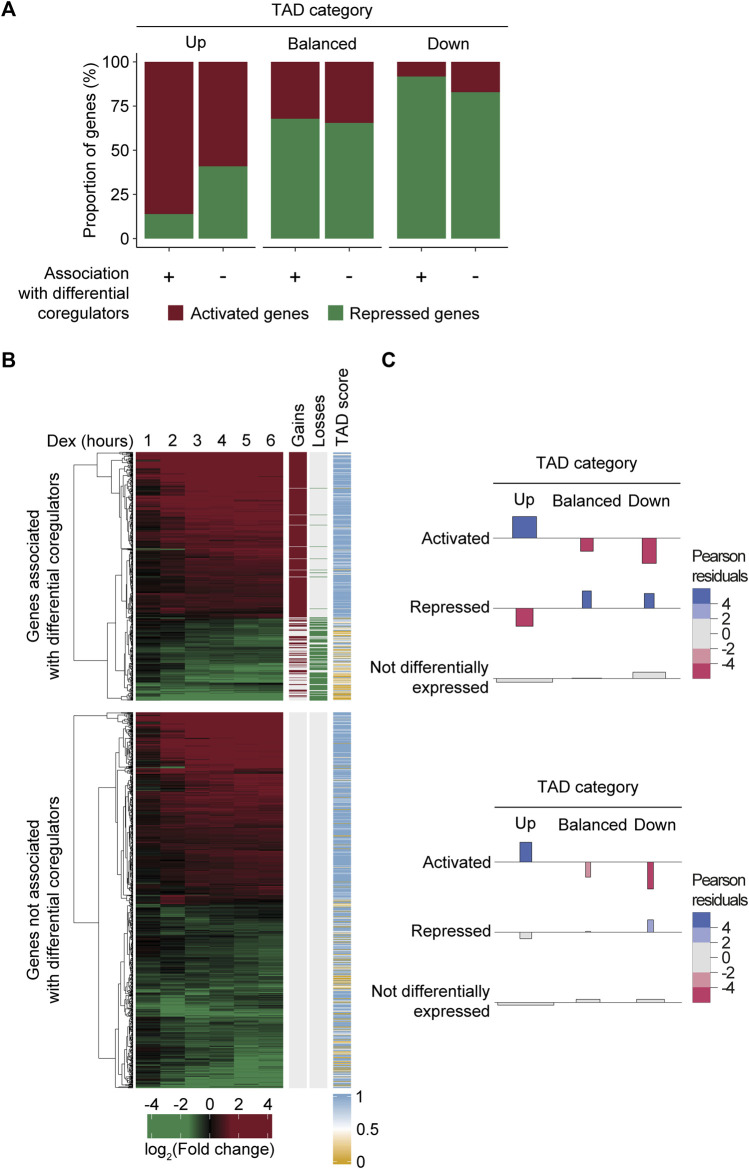
Dex-regulated genes are enriched within TADs matching their activity independently from the differential recruitment of MED1 and BRD4. **(A)** Dex-regulated genes associated with the differential recruitment of MED1 and BRD4 or not were found within TADs matching their activity. Dex-activated and -repressed genes were assigned TADs and subdivided whether they were associated to a region with the differential recruitment of transcriptional coregulators (*n* = 532) or not (*n* = 805). Data are represented as a percentage of the total number of genes. **(B)** Representation of the 1,337 Dex-regulated genes found within a responsive TAD. RNA levels in the fold change (log2) are displayed from 1 to 6 h after Dex stimulation. Hierarchical clustering (using the Euclidean distance) was applied to the gene expression matrix and is represented by the dendogram. Regions gaining or losing MED1 and BRD4 are represented by red and green lines, respectively. TAD scores were calculated and are represented as mentioned before. **(C)** Association plot illustrating the dependence between changes at the RNA level for Dex-regulated genes and the category of the TAD (up, balanced, and down). The height of each bar is proportional to the Pearson residual, while the width is proportional to the square root of the expected frequency so that the area of the rectangle is proportional to the difference between observed and expected frequencies. Residual values are colored if greater than 2 (enrichment, blue) or less than −2 (depletion, red). Top—Dex-regulated genes associated with the differential recruitment of MED1 and BRD4. Bottom—Dex-regulated genes not associated with the differential recruitment of MED1 and BRD4.

While regions gaining and losing MED1 and BRD4 were strongly associated with variations in gene levels, not all Dex-regulated genes were associated with the differential recruitment of transcriptional coregulators. Indeed, the fact that 60% and 78.9% of activated and repressed genes following Dex treatment were not associated with differential coregulators raised questions about the mechanisms involved. We hypothesized that the activity of the TAD environment of a gene was an important determinant of gene level variations in addition to the differential recruitment of transcriptional regulators by the GR. The majority of 1,759 Dex-regulated genes (76%) were enriched within a TAD, gaining or losing MED1 and BRD4 (permutation test, *n* = 10,000, and *p* = 9.99e-5) (Supplementary Table S3). As expected, activated and repressed genes were enriched within TADs scored as up (Fisher’s test, OR = 5.02, and *p* = 7.56e-113) and down (Fisher’s test, OR = 2.07, and *p* = 7.79e-14). If the TAD environment surrounding a gene is an important determinant in the response to Dex, differentially expressed genes should be found within up and down TADs, matching the changes independently from the differential recruitment of transcriptional coregulators. Globally, 39.8% of Dex-regulated genes assigned to a responsive TAD were associated with a region gaining or losing MED1 and BRD4, compared to 60.2% that were not (Supplementary Table S3). Interestingly, whether the differentially expressed genes were associated to regions gaining or losing MED1 and BRD4 or not, they were enriched within a TAD matching their activity (Figures 4A, B). Indeed, chi-squared tests revealed a significant association between the direction of the gene response and the activity of the TAD, whether the gene was occupied (χ^2^ = 214.42 and *p* = 2.98e-45) or not (χ^2^ = 81.58 and *p* = 8.07e-17) by regions gaining or losing MED1 and BRD4 (Figures 4B, C). Analysis of Pearson residuals using a critical absolute value of 2 showed that Dex-regulated genes associated with the differential recruitment of MED1 and BRD4 or not were enriched within a TAD category matching the gene activity (Figure 4C). To validate our observations, we subdivided Dex-regulated genes into bound or not by the GR. Statistically significant relationships between the direction of the gene response and the activity of the TAD, whether the gene was bound by the GR (χ^2^ = 163.69 and *p* = 2.36e-34) or not (χ^2^ = 71.24 and *p* = 1.24e-14), were confirmed (Supplementary Figure S3). These results suggest that, in the absence of a measurable differential recruitment of transcriptional coregulators and GR binding, the presence of a gene within a specific TAD is an important determinant of the response.

### The TAD environment has less impact than the recruitment of the GR and transcriptional coregulators

**FIGURE 5:**
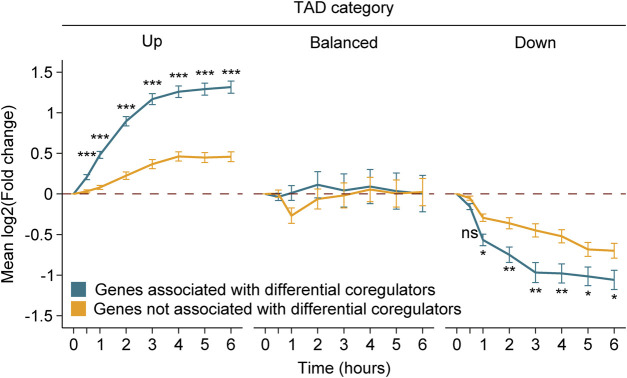
Differential recruitment of MED1 and BRD4 translates into a stronger gene response. Gene level variations match the transcriptional activity of the TAD following Dex treatment. Genes associated to a region with the differential recruitment of MED1 and BRD4 (blue) or not (yellow) are displayed up to 6 h after treatment. For each TAD category, mean variations in RNA levels through time and the standard error of the mean (error bars) are represented. Mann–Whitney U tests were used to compare RNA level variations between genes with and without the differential recruitment of transcriptional coregulators at 0.5, 1, 2, 3, 4, 5, and 6 h. The Benjamini–Hochberg procedure was applied on the empirical *p*-values to correct for multiple testing. ns, not significant; *p < 0.05, **p < 0.01, and ***p < 0.001.

Our results support that the differential amount of MED1 and BRD4 is associated with changes at the gene level (Figure 2). If true, differentially expressed genes following Dex stimulation that are influenced by the environment of the TAD should be less affected compared to those associated with regions gaining and losing MED1 and BRD4. To determine if the amplitude of the differential gene regulation was equivalent between genes associated or not with regions gaining or losing MED1 and BRD4, we evaluated the trajectories of gene expression changes throughout the 6-h time course for each category of TAD (up, balanced, and down) (Figure 5). As expected, global gene activity was correlated with the TAD classification, and gene activation or repression was observed in TADs gaining or losing MED1 and BRD4, respectively. The contact with regions gaining or losing transcriptional coregulators was associated with stronger activation (between 2.73- and 6.51-fold increases) and repression (between 1.49- and 2.16-fold decreases). Similar results were obtained when differentially occupied genes were subdivided in bound or not by the GR with the exception that no differences were observed between repressed genes (Supplementary Figure S4). Taken together, these results show that while primary DNA binding by a transcription factor and the differential recruitment of transcriptional coregulators produce a stronger gene response, the surrounding TAD environment also influences the gene output.

## Discussion

### Model for regionalized transcriptional regulation

Our results establish that following ligand activation, the GR elicits regionalized gains and losses in transcriptional coregulators MED1 and BRD4, creating favorable or unfavorable environments for transcriptional regulation. The primary consequence of the GR recruitment is to modulate levels of transcriptional coregulators, which not only leads directly to the activation or repression of the bound target genes but also influences other genes in the vicinity (Figures 1, 4). While we cannot exclude the possibility that genes not bound by the GR and not associated with regions gaining or losing MED1 and BRD4 are false negatives due to technical reasons, we envision two possibilities to explain how a regionalized effect on transcriptional regulation is achievable. On one hand, phase separation droplets created by the accumulation of transcription regulators could diffuse and be used as cargo between genes (Silveira and Bilodeau, 2018). Accordingly, modulation of the local concentration of transcriptional regulators could facilitate or hamper their diffusion to neighboring genes and affect transcription. This model is supported by the GR implication in the formation of biological condensates during gene activation (Stortz et al., 2020; Frank et al., 2021). On the other hand, modifying the recruitment of transcriptional regulators could impact the dynamic of the regulatory regions themselves and the frequency of contact with promoters within a TAD. This model is supported by the existence of subdomains or chromatin modules within TADs characterized by short-range enhancer–promoter and promoter–promoter interactions and the increased dynamics of regulatory regions after hormonal stimulation (Le Dily and Beato, 2018; Hsieh et al., 2020; Krietenstein et al., 2020; van Mierlo et al., 2023). The low resolution of Hi-C approaches can explain why we failed to detect physical interactions between unbound differentially expressed genes and GR-occupied regions. Be that as it may, our results globally support regional transcriptional consequences for genes in proximity to regulatory regions responding to a transcription factor, whether binding or physical interactions are detected.

### Implication of the model for gene positioning effects in mammalian cells

The proposed model represents an important shift in the conception of direct and indirect effects elicited by transcription factors. Current methods to determine the primary effects of a transcription factor are based on the integration of multi-omics data. For example, chromosome architecture data are coupled to genome-wide localization of the transcription factor to determine transcription factor-bound regions and assign them to genes. Following the integration of gene expression datasets, differentially expressed genes that are unbound are typically discarded and labeled as indirect effects. Here, we are proposing that recruitment of a transcription factor and its associated coregulators has two types of primary effects on transcription: 1) a direct effect through DNA binding of the transcription factor at cis-regulatory regions and 2) a domain-dependent effect based on the position in a specific environment. It is important to note that what we are describing as domain-dependent could represent highly dynamic transcription factors or physical contacts with cis-regulatory regions making detection of enhancer–promoter contacts more difficult. The distinction between these models and the precise definition of the boundaries in which they are acting will require further investigations. Nonetheless, our interpretation is an important distinction from the current definition of indirect effects. Based on our assessment, inducing a transcription factor will redistribute transcriptional coregulators, leading to primary direct and indirect effects. With this nomenclature, gene expression changes independent of direct DNA binding or position effects would be considered as secondary.

### Distinction between gene activation and repression

Transcription factors activate and repress genes by modulating recruitment of coregulators (Lambert et al., 2018). Our results support that gains in transcriptional coregulators MED1 and BRD4 are dependent on the presence of the GR on chromatin. However, for most regions where losses in MED1 and BRD4 were observed, the GR was not detected (Supplementary Table S2). While the presence of the GR at repressed regions could be harder to detect, the result also suggests that the recruitment of the GR at chromatin is not necessary to remove transcriptional coregulators. The GR was shown to titrate and sequester transcriptional coregulators, suggesting a passive mechanism of gene repression referred to as squelching (Schmidt et al., 2016; Bothe et al., 2021; Portuguez et al., 2022). Considering that genes repressed by the GR are active in basal conditions, repression can be driven by opportunity. When the concentration of the GR increases in the nucleus, interference with the transcriptional program in place would provide the transcriptional coregulators required for subsequent transcriptional activation. This interpretation is supported by the fact that higher levels of MED1 and BRD4 are found at Dex-repressed genes compared to Dex-activated genes in normal conditions (Portuguez et al., 2022). This type of passive mechanism would result in repression being distributed throughout active genes rather than being targeted. This model would explain why a limited number of regions with a significant loss in MED1 and BRD4 were identified (Supplementary Table S2). Interestingly, GR-responsive genes share spatial domains specialized in activation or repression (Portuguez et al., 2022). Whether activation domains require spatial association to a repressed domain is an open question. Interestingly, while GR has been extensively studied molecularly, no mutant with the ability to activate transcription without also repressing has previously been reported to our knowledge (the ability of mutants to repress without activating is frequent) (Beck et al., 2011). It will be interesting to determine if repression is mandatory for the ability of the GR to activate transcription.

## Conclusion

The influence of the genomic microenvironment has been associated with the gene position effect in mammalian cells. We are proposing that TADs are being modulated by the redistribution of transcriptional coregulators. Therefore, when a transgene is added to different genomic contexts, access to mobile transcriptional regulators or dynamic cis-regulatory regions will differ. Furthermore, this model explains the fast kinetics of differential expression observed for genes not bound by an induced transcription factor but responding to stimulation. It remains to be determined if, during the biological response of a cell, activation and repression mechanisms are molecularly linked.

## Data Availability

The data generated for this publication is available on GEO (Edgar et al., 2002) under accession number GSE226487. The first replicate for the whole cell extract (GSM2040031) and MED1 (GSM2040033) in control conditions matching their Dex-stimulated counterparts are already available under accession number GSE76893. The ChIP-seq data are viewable on the UCSC genome browser here: https://genome.ucsc.edu/s/ckntav/A549_Dex_response. All publicly available sequencing datasets used in the manuscript are listed in Supplementary Table S5.

## Supplementary material

SUPPLEMENTARY FIGURE S1 Dex-dependent changes at the RNA and chromatin levels. **(A)** Gene level variations for *ANGPTL4* and *IL11* genes following Dex stimulation. Variations in RNA levels through time were determined after smoothing the trajectories using the local regression (LOESS) method. **(B)** RNA-seq profiles of *ANGPTL4* and *IL11* genes following Dex stimulation. Tracks are displayed in reads per kilobase per million mapped reads (RPKM). Gene depictions are presented below the RNA-seq tracks. **(C)** Scatter plot displaying the difference in normalized count values between Dex and control conditions for MED1 and BRD4. Each dot represents a genomic region defined as differentially recruiting transcriptional coregulators. Correlations were assessed using Pearson’s correlation method. **(D)** Motif enrichment analysis of genomic regions gaining (top) or losing (bottom) MED1 and BRD4. The top three DNA-binding motifs recovered using the JASPAR database are displayed.

SUPPLEMENTARY FIGURE S2 Differential recruitment of MED1 and BRD4 is associated with the gene response to Dex. **(A)** Quantification of the number of Dex-regulated genes after 0.5, 1, 2, 3, 4, 5, and 6 h of stimulation. Activated (red) and repressed (green) genes are represented at each time point. **(B)** Scatter plots showing the correlation between variations in RNA levels of Dex-regulated genes and the recruitment of transcriptional coregulators at their cis-regulatory regions after 0.5, 2, 3, 4, 5, and 6 h. For RNA levels, the log2 of the fold change is represented. For recruitment of MED1 and BRD4, the log2 of the read density at each differential region is represented. Regions gaining and losing MED1 and BRD4 were associated to genes based on their linear proximity or the presence on 3D chromatin interactions. Correlations were assessed using Pearson’s correlation method.

SUPPLEMENTARY FIGURE S3 Dex-regulated genes are enriched within TADs matching their activity independently from GR binding. **(A)** Dex-regulated genes bound by the GR or not were found within TADs matching their activity. Dex-activated and -repressed genes were assigned TADs and subdivided whether they were associated to a region with the differential recruitment of the GR (*n* = 857) or not (*n* = 480). Data are represented as a percentage of the total number of genes. **(B)** Representation of the 1,337 Dex-regulated genes found within a responsive TAD. RNA levels in fold change (log2) are displayed from 1 to 6 h after Dex stimulation. Hierarchical clustering (using the Euclidean distance) was applied to fold changes and is represented by the dendogram. Regions gaining or losing MED1 and BRD4 are represented by red and green lines, respectively. TAD scores were calculated and are represented as before. **(C)** Association plot illustrating the dependence between changes at the RNA level for Dex-regulated genes and the category of the TAD (up, balanced, and down). The height of each bar is proportional to the Pearson residual, while the width is proportional to the square root of the expected frequency so that the area of the rectangle is proportional to the difference between observed and expected frequencies. Residual values are colored if greater than 2 (enrichment, blue) or less than -2 (depletion, red). Top—Dex-regulated genes with GR binding. Bottom—Dex-regulated genes without GR binding.

SUPPLEMENTARY FIGURE S4 Differential binding of the GR translates into stronger gene activation. Genes associated to GR binding (blue) or not (yellow) are displayed up to 6 h after treatment. For each TAD category, mean variation in RNA levels through time and the standard error of the mean (error bars) are represented. Mann–Whitney U tests were used to compare RNA level variations between genes with and without GR binding at 0.5, 1, 2, 3, 4, 5, and 6 h. The Benjamini–Hochberg procedure was applied on the empirical *p*-values to correct for multiple testing. ns, not significant; **p* < 0.05 and ****p* < 0.001.

SUPPLEMENTARY TABLE S1 List of genomic regions for MED1 and BRD4 ChIP-seq datasets in control and Dex-stimulated conditions.

SUPPLEMENTARY TABLE S2 List of genomic regions with the differential recruitment of MED1 and BRD4 following Dex stimulation.

SUPPLEMENTARY TABLE S3 List of Dex-regulated genes.

SUPPLEMENTARY TABLE S4 List of genomic coordinates for TADs.

SUPPLEMENTARY TABLE S5 List of publicly available datasets used in this study.
