# Supplementary figures and images for: Glucocorticoid stimulation induces regionalized gene responses within topologically associating domains
Source: Front Genet. 2023 Jul 27;14:1237092. doi: 10.3389/fgene.2023.1237092 (PMC10413275; doi:10.3389/fgene.2023.1237092)

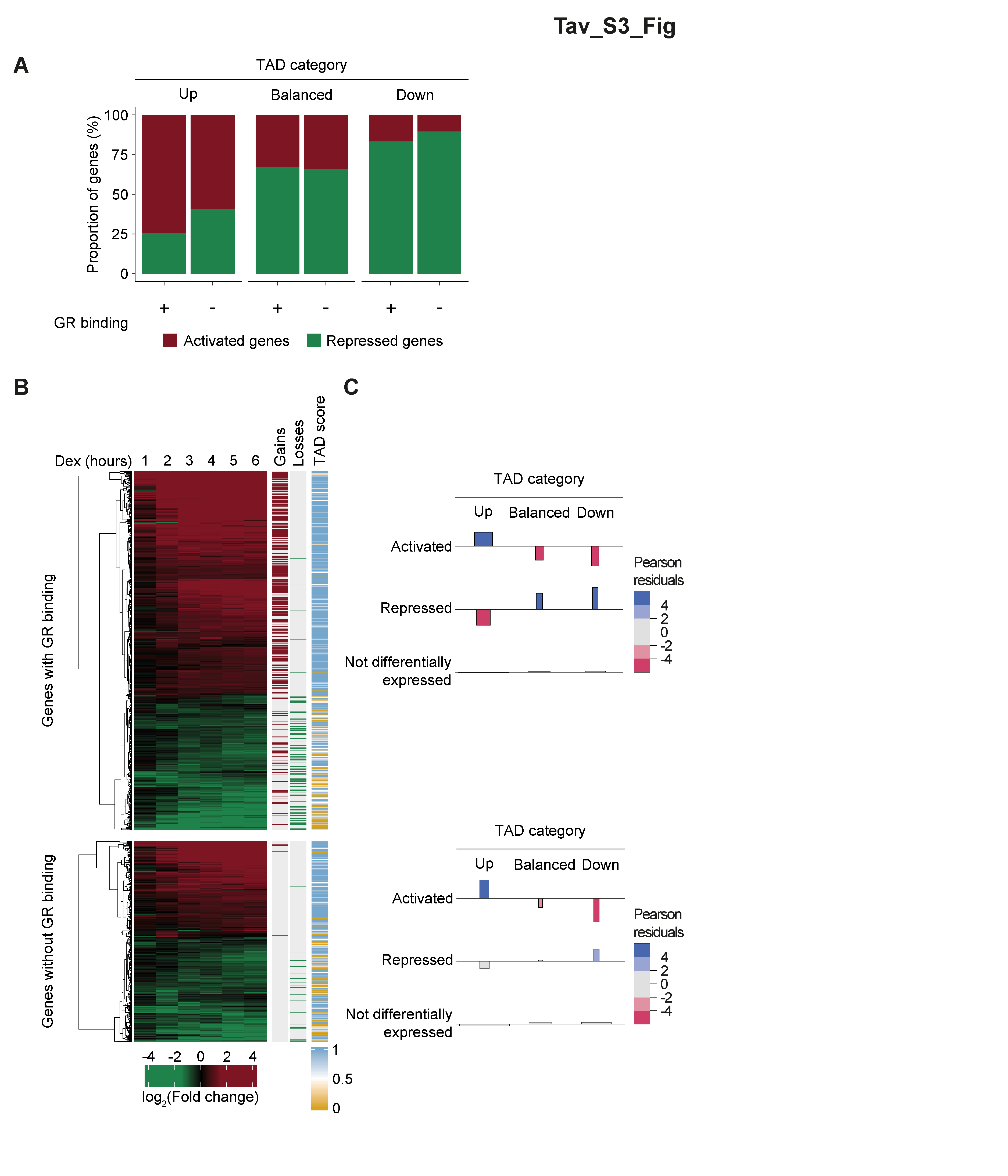

Supplement: Supplementary file 3 [file Image3.tif]

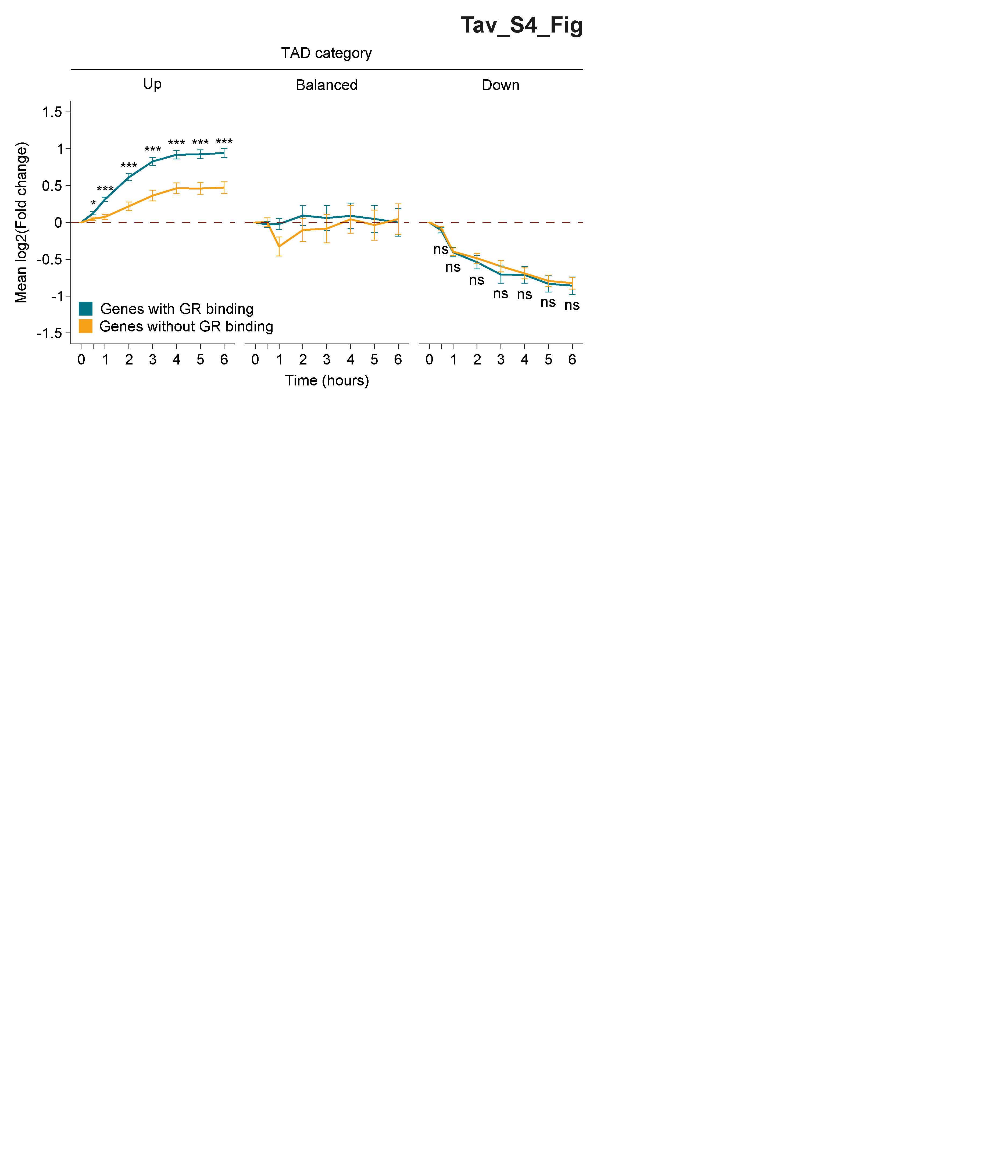

Supplement: Supplementary file 4 [file Image4.tif]

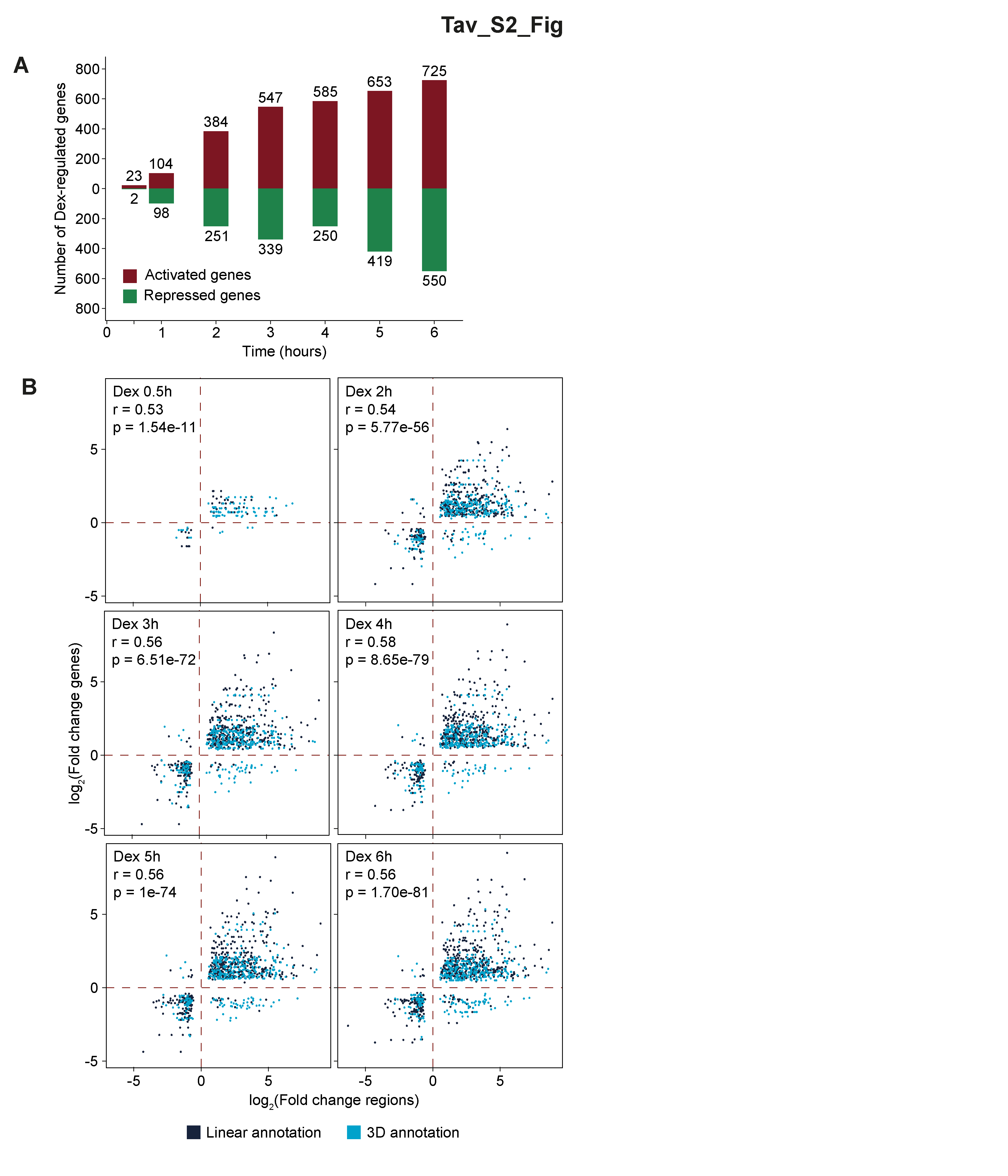

Supplement: Supplementary file 5 [file Image2.tif]

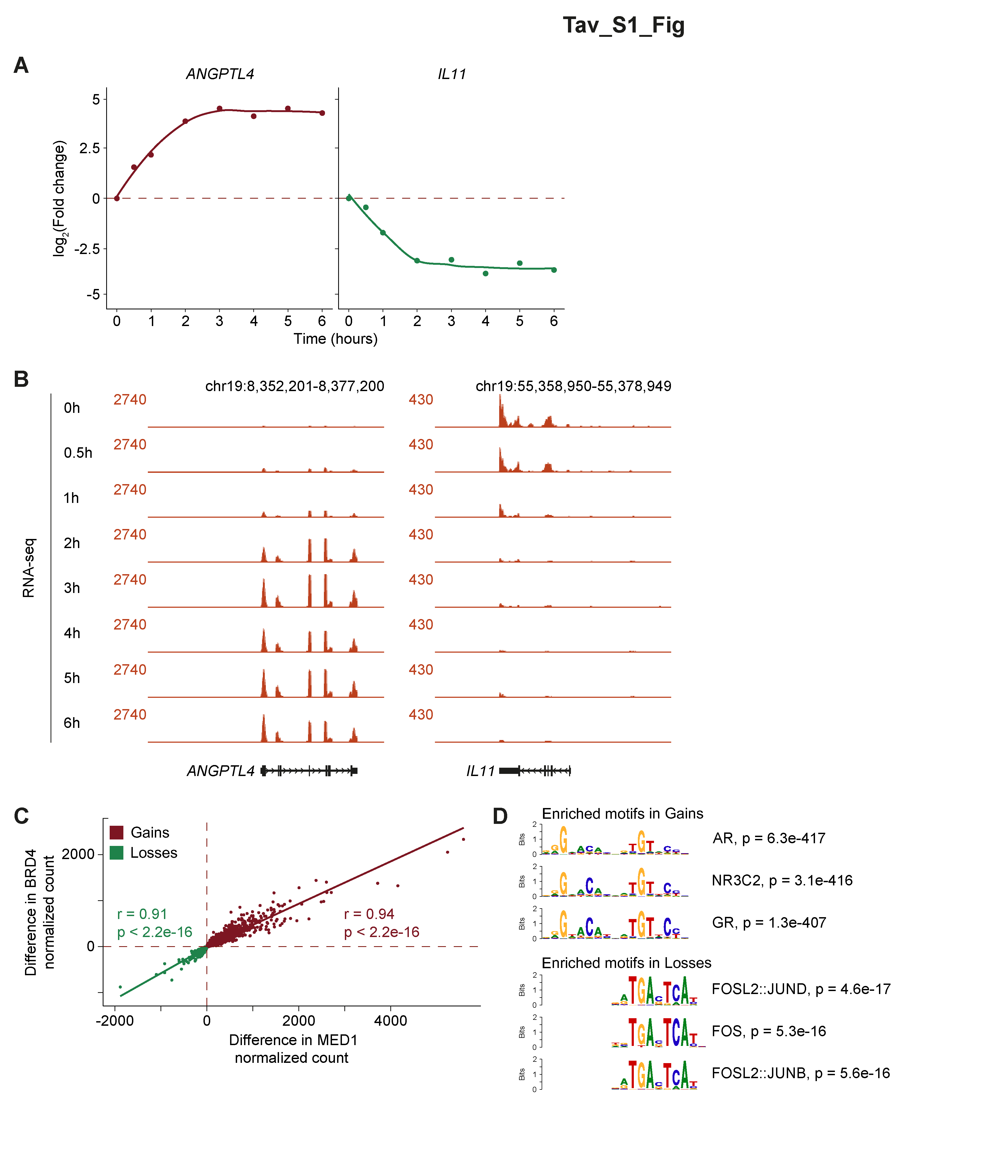

Supplement: Supplementary file 6 [file Image1.tif]
